# Learning from aviation safety: a call for formal "readbacks" in surgery

**DOI:** 10.1186/1754-9493-2-21

**Published:** 2008-09-17

**Authors:** Philip F Stahel

**Affiliations:** 1 Department of Orthopaedic Surgery, Denver Health Medical Center, University of Colorado School of Medicine, 777 Bannock Street, Denver, CO 80204, USA

## 

The first fatal airplane crash in history occurred exactly 100 years ago, on September 17, 1908, when Army lieutenant Thomas Selfridge died in a failed flight attempt with the aviation pioneer Orville Wright. Since that time, aviation safety standards have significantly improved. Currently, the risk for an American dying in an airplane crash is about 1:500,000, compared to a 1:20,000 chance of dying in a car accident. In the field of medicine, it was not until the shocking report by the *Institute of Medicine *in 1999 revealed that 100,000's of patients die in the United States every year as a consequence of medical errors [1], when we began to realize that there is something "wrong with the system". While this unacceptably high number has been chronically underrated in public recognition, an extrapolation of these statistics to professional aviation equals to about 200 jumbo jet crashes per year, or one 747 crash every other day. This dramatic insight led to the design of the "100,000 lives campaign" by the *Institute for Healthcare Improvement *in 2004 [2]. By 2006, the campaign had surpassed its initial goal by saving more than 120,000 lives through the implementation of increased patient safety standards and algorithms [2]. These include the recent implementation of a standardized surgical *"time-out" *to ensure the correct patient identity and correct procedure performed at the correct surgical site [3]. In addition, the implementation of formal, structured perioperative briefings in the operating room have been shown to significantly reduce the incidence of wrong site surgeries [4].

Despite those recent improvements, the analysis of the *American College of Surgeons*' closed claims study revealed that a breakdown in communication before, during, or after surgery still represents a significant source of errors which lead to patient complications [5]. Of these, 85% of adverse events related to communication breakdown occurred by verbal communication, while only 4% were attributed to communication in written form [5]. This notion provides the basis for a call for written checklists and formal verbal "readback" orders among healthcare professionals who care for surgical patients, in order to avoid or reduce the high incidence of perioperative complications related to a breakdown in communication. Interestingly, pilot readbacks represent a hallmark safety concept in professional aviation. While the current debate in aviation safety is related to optimizing and correcting the modality of readbacks [6,7], this crucial form of communication is still virtually nonexistent among surgeons. Dr. Eddie Hoover has characterized the issue to the point, in a recent editorial: *"Getting surgeons to readback orders and instructions will age you 10 years, yet the Navies of the world have demonstrated for eons that it improves efficiency, promotes safety, and saves lives." *[8].

I wish to emphasize that the implementation of verbal readback orders represents the 2^nd ^National Patient Safety Goal (NPSG) for 2009, as defined by the Joint Commission [9]. The NPSG #02.01.01, aimed at improving the effectiveness of communication among caregivers, is defined as such: *"For verbal or telephone orders or for telephone reporting of critical test results, the individual giving the order or test result verifies the complete order or test result by having the person receiving the information record and 'read back' the complete order or test result." *[9].

In conclusion, I urge all healthcare professionals involved in the care of surgical patients to contribute to improved patient safety and reduced complications and sentinel events in 2009 by addressing the most frequent root cause for adverse outcome in surgery: Ineffective communication. The implementation of formal standardized "readbacks" is a promising start.

## Competing interests

The author declares that he has no competing interests.
